# Early Left-Hemispheric Dysfunction of Face Processing in Congenital Prosopagnosia: An MEG Study

**DOI:** 10.1371/journal.pone.0002326

**Published:** 2008-06-04

**Authors:** Christian Dobel, Christian Putsche, Pienie Zwitserlood, Markus Junghöfer

**Affiliations:** 1 Institute for Biomagnetism and Biosignalanalysis, Münster University Hospital, Münster, Germany; 2 Otto Creutzfeldt Center, Westfälische Wilhelms-Universität Münster, Münster, Germany; 3 Department of Psychology, Westfälische Wilhelms-Universität Münster, Münster, Germany; University of Minnesota, United States of America

## Abstract

**Background:**

Congenital prosopagnosia is a severe face perception impairment which is not acquired by a brain lesion and is presumably present from birth. It manifests mostly by an inability to recognise familiar persons.

Electrophysiological research has demonstrated the relevance to face processing of a negative deflection peaking around 170 ms, labelled accordingly as N170 in the electroencephalogram (EEG) and M170 in magnetoencephalography (MEG). The M170 was shown to be sensitive to the inversion of faces and to familiarity-two factors that are assumed to be crucial for congenital prosopagnosia. In order to locate the cognitive dysfunction and its neural correlates, we investigated the time course of neural activity in response to these manipulations.

**Methodology:**

Seven individuals with congenital prosopagnosia and seven matched controls participated in the experiment. To explore brain activity with high accuracy in time, we recorded evoked magnetic fields (275 channel whole head MEG) while participants were looking at faces differing in familiarity (famous vs. unknown) and orientation (upright vs. inverted). The underlying neural sources were estimated by means of the least square minimum-norm-estimation (L2-MNE) approach.

**Principal Findings:**

The behavioural data corroborate earlier findings on impaired configural processing in congenital prosopagnosia. For the M170, the overall results replicated earlier findings, with larger occipito-temporal brain responses to inverted than upright faces, and more right- than left-hemispheric activity. Compared to controls, participants with congenital prosopagnosia displayed a general decrease in brain activity, primarily over left occipitotemporal areas. This attenuation did not interact with familiarity or orientation.

**Conclusions:**

The study substantiates the finding of an early involvement of the left hemisphere in symptoms of prosopagnosia. This might be related to an efficient and overused featural processing strategy which serves as a compensation of impaired configural processing.

## Introduction

Investigating individuals who show an impairment in the processing of faces has proved to be beneficial for the general understanding of such processes in the normal, unimpaired case. Since the initial studies of Bodamer [1], the condition in which subjects show a weakness in face processing is referred to as prosopagnosia. Such a condition usually arises as a consequence of lesions in extrastriate occipitotemporal areas of the right, or of the right and left hemisphere, and is labelled acquired prosopagnosia [see e.g. 2]–[3]. After the first reports by Bodamer, several case studies followed and acquired prosopagnosia was studied intensively [e.g. 4]–[6].

In recent years, evidence has been accumulated which suggests that prosopagnosia not only arises as a consequence of brain lesions. It may also exist from early childhood onwards, without a known incidence that triggered the impairment. For this reason such cases were labelled as “developmental” (emphasizing the early origin; note, however, that cases are subsumed under “developmental” for which an early brain damage has been reported. For that reason we preferred the alternative term “congenital” even though so far it has not been proven that the impairment actually exists from birth) or “congenital” (emphasizing the inborn nature) prosopagnosia. There is increasing evidence that this latter type of impairment runs in families [7]–[9], and it seems to be especially prevalent in patients suffering from Asperger syndrome and other social-emotional disorders [10]. It is a matter of an ongoing and intense debate whether the impairment is only restricted to the processing of faces, or whether it is accompanied by more general deficits of visual processing. On the one hand, clear dissociations between intact object processing and reduced face processing abilities were described [11] with most of the prosopagnosic participants performing within the normal range in several object recognition tests. Moreover, in a single case-study, a person suffering from congenital prosopagnosia was well able to learn to differentiate similar exemplars from a novel animal-like object category [“greebles”; 12]–[13]. On the other hand, a severe object agnosia can occur without affecting face perception and recognition, suggesting a double dissociation between object and face perception at a neuronal level [14]–[15]. Such cases and arguments strengthen the assumption that first, face recognition is subserved by face-specific cognitive and neural mechanisms [for a recent overview see ref. 16] and that second, congenital prosopagnosia is not inevitably associated with object-recognition deficits.

However, two recent group studies demonstrated that individuals suffering from congenital prosopagnosia also show impairments in other perceptual domains, if more ‘global’ instead of ‘local’ processing strategies are required. With global-local stimuli (large letters constructed from small letters, such as large E made of small Ks), normal subjects show a global precedence effect: They are faster at detecting the global than the local letters. In local-letter detection, the global letter interferes, but not vice versa. Prosopagnosics show local rather than global interference, as they tend to respond more slowly to global than to local letters [17]–[18]. Resuming the results of behavioural studies, there seems to be an agreement that individuals suffering from congenital prosopagnosia show a deficit in global or configural processing and a preference for the processing of local (or featural) information. Configural information is to be broadly understood as concerning the spatial relationships of facial features (e.g. eyes, nose and mouth) [for an overview of definitions see ref. 28].

Configural processing in face processing is most often investigated by comparing responses to upright and inverted faces, which typically results in the so-called face inversion effect [for an overview see ref. 19]. Slower responses and higher error rates are associated with the identification of inverted as compared to upright faces [20]. Interestingly, efficient and rapid recognition of inverted faces cannot be learned, even with over 1000 repetitions of the same inverted face [21]. In contrast, inversion learning easily occurs for other types of familiar objects [22]. The face inversion effect is a well established finding and became a hallmark for the assumption of specialized face processing. Interestingly, some studies were unable to find a face inversion effect in at least some individuals with acquired prosopagnosia [23]–[25], and some with congenital prosopagnosia [13], [26]–[27]. The debate about the exact cognitive locus of the impairment will certainly continue, presumably also because the differences between configural, holistic and global processing are not yet clear. As Maurer *et al.* [28] pointed out, several authors subsume holistic processing under configural processing. Nevertheless, it seems safe to say that inversion of faces hampers fast and almost effortless recognition, and thus has a clear impact on configural/holistic/global processing.

The present study investigates configural processing by contrasting the perception of known and unknown faces in upright and inverted orientation in congenitally prosopagnosic and control participants. We chose magnetoencephalography as dependent measure, as it allows to track neural responses with highest temporal and fair spatial resolution. In the following, we provide a short introduction to the main ERP (event related potential) components investigated in face perception research, and summarize the current findings on neurophysiological correlates of congenital prosopagnosia.

Event related potentials are widely used to investigate the neural mechanisms of face perception [for an overview see ref. 29]. In particular, the N170 component received and receives much interest, as it was originally thought to exclusively reflect neural processing of faces or face parts [30]. More recent studies, however, revealed that, rather than being face specific, the N170 component generally arises in the presence of visually homogenous stimuli for which subjects possess some expertise [31]–[32]. Nevertheless, there is general consensus that this component is a crucial index for early processes of face perception. The N170 seems to reflect the process of structural encoding, resulting in a pre-categorical representation of faces which is subsequently used to access stored representations of familiar faces [33].

Results of ERPs studies on face perception in both acquired and congenital prosopagnosia are mixed. Eimer and McCarthy [34] reported a patient with acquired prosopagnosia who showed no N170 in response to both upright and inverted faces, while a patient studied by Bobes and colleagues displayed clear N170 effects [35]. Single-case studies with congenital prosopagnosics suggest that their N170 lacks specificity for faces, in contrast to objects [18], [36]–[37]. Interestingly, prosopagnosics also demonstrated impaired configural processing evidenced by a smaller N170 component not only in response to faces, but also to bodies [38]. It was concluded, that both face and body perception rely on a shared network stressing the involvement of configural processing in other stimulus categories besides faces.

Mixed results were observed in a small-group study of five congenital prosopagnosics. A non-discriminative M170 (the neuromagnetic counterpart of the N170) was observed in three subjects, and a robust discriminative M170 in two others [39]. Similarly, Minnebusch and colleagues [40] found reliable N170 differences for faces compared to non-face stimuli in three of four individuals with congenital prosopagnosia. These rather heterogeneous findings confirm that congenital prosopagnosia is most likely not a homogenous impairment, as also evidenced in recent behavioural group studies [9], [41]. They also emphasize that face processing deficits are not necessarily correlated with the lack of a face-specific N170.

With respect to the neural substrate of face processing, there is overwhelming evidence from functional magnetic resonance imaging (fMRI) and intracranial electrophysiological recording that the Fusiform Face Area (FFA) is activated (mostly bilaterally, but more in the right hemisphere) in unimpaired face perception [42]–[44]. It is astounding that the majority of studies report normal FFA activation in individuals with congenital prosopagnosia [45]–[46]. A recent study comparing fMRI and ERPs in one prosopagnosic subject demonstrated normal hemodynamic FFA activity, but no ERP selectivity for faces [18]. One congenital prosopagnosic studied by Hadjikhani and de Gelder [47] showed no differentiation in FFA activity between faces, houses, and other objects (although some FFA dysfunction was found for individuals with acquired prosopagnosia). Recent fMRI data by Steeves and colleagues [48], from a subject with acquired prosopagnosia, showed inconspicuous and face-selective activation of the FFA. Together, these data clearly suggest that normal hemodynamic FFA activation does not necessarily result in successful overt face recognition. Nevertheless, based on morphometric and volumetric analyses, Behrmann and colleagues [49] suggested the anterior fusiform gyrus as a potential neuroanatomic locus for congenital prosopagnosia. The volume reduction in this area correlated significantly with recognizing famous faces, but not with face discrimination. Consequently, the comparison of known and unknown faces seems to be the proper test to tap into the neural correlates of congenital prosopagnosia.

To sum up, there is some agreement that congenital prosopagnosia results from impairment of configural/global processing, even though this might not be exclusive for faces. In order to differentiate individuals with congenital prosopagnosia from controls, the use of electrophysiology with high temporal resolution seems more fruitful than hemodynamic methods. Since hemodynamic changes reflect the integrated neural activity within seconds, fMRI and PET may not be sensitive enough to track the possibly rapid and transient neuronal correlates of this disorder, and may not be able to separate early effects from later, possibly compensatory processes in identical brain areas.

We thus used whole head magnetoencephalography (MEG) to investigate the time course of neural activation in response to faces in individuals with congenital prosopagnosia and matched control subjects. Because the majority of studies compared face and object processing in prosopagnosia, rather little is known about the brain response to known and unknown faces, and to face inversion, in congenital prosopagnosia. In a study with unimpaired participants, we demonstrated [50] that the M170 is sensitive to face inversion and familiarity. For the current study, we predict effects of familiarity, possibly showing up as an interaction between familiarity and inversion. We also expect differences between prosopagnosics and controls. Because of their local precedence, we also assume that people suffering from congenital prosopagnosia show a reduced inversion effect. On the other hand, if face processing is subserved by modules specialized for faces [51], this should be expressed by overall less activity in prosopagnosics. In the light of their face-recognition problems, we expect reduced or no ERF (event related field) differentiations between known and unknown faces. Given the results from the current literature, these electrophysiological differences should appear most strongly over areas of the right hemisphere.

## Methods

### Participants

We investigated seven individuals suffering from congenital prosopagnosia and seven controls that were matched for age (controls: 39 years mean age (25–57); prosopagnosics: 38 years mean age 38 (22–57)), sex (in both groups: 4 men and 3 women), handedness (all right-handed) and education (high-school diploma). All controls were well known by at least one of the authors, and none reported any problems with face perception. The group of congenital prosopagnosics was tested with a battery of neuropsychological tests and experiments, described in detail in [9]. Several standardized tests were administered, such as the Visual Object and Space Perception battery (VOSP) [52], and the Bielefelder Famous Faces Test (BFFT) [53]. The congenital prosopagnosics showed inconspicuous results for the general visual functions tested, including all tests of the VOSP. Regarding face perception, all prosopagnosics showed fundamental impairments in recognizing famous people if based on visual cues only. This was not due to lack of knowledge, since they recognized as many famous people as controls when provided with verbal cues. In addition, it took the prosopagnosic subjects much longer than controls to respond to faces compared to eye-glasses in a delayed matching to sample task. Based on these results, we took these two tests as main diagnostic criteria for congenital prosopagnosia. Other tests for functions of face perception such as judging age, emotion or gender were not discriminative between the two groups. Four of the prosopagnosics MH, GH, XG and XS were reported in detail in [9]. BT and LO were also investigated with the test battery of [9] and showed similar results which will be published elsewhere. KA is a 28 year old, male physician who was not reported before. As the remaining individuals with congenital prosopagnosia, he performed inconspicuously in the tests on object recognition. With respect to tests on face perception, his performance was comparable to the remaining prosopagnosics. He recognized 36% of the famous faces in the BFFT by visual cues only, but 86% based on verbal cues. His latency difference between faces and glasses in the delayed matching to sample task was more than two standard deviations away from the mean of controls.

All participants gave their written consent to participate in the study. The study falls under the ethical approval of the “Kommission der Ärztekammer Westfalen-Lippe und der Medizinischen Fakultät der Westfälischen-Wilhelms Universität Münster”.

### Stimulus Material

Stimuli consisted of pictures taken from the faces of 66 famous people with regular appearance in the German media, and 66 faces unknown to the participants. These pictures were also used in an earlier study [50]. Twenty additional facial stimuli served as practice trials. The selection of famous persons was done in a pilot study, and pictures of famous faces were only included if they were recognized by at least 15 of the 21 pilot control subjects (>70%). Across categories, faces were matched for age and gender, and all faces showed neutral emotional expressions. Using Adobe Photoshop® pictures were edited, replacing the background by a uniform grey, leaving only face and hairline. All pictures were converted to greyscale, with a size of 6 degrees of visual angle in height. The sets of famous and unknown faces did not differ with respect to overall brightness (t(130) = −1.49; n.s.) and contrast (t(130) = .34; n.s.). Each picture was presented in upright and an inverted position.

### Experimental procedure

MEG recording took place in a dimly lit, sound-attenuated and magnetically shielded chamber. Stimuli were projected by means of a mirror system onto a screen, with a viewing distance of 57 cm. Individual head shapes and three landmark coils, attached to the two auditory canals and the nasion were digitized using a Polhemus 3Space® Fasttrack prior to the measurement in order to determine the head coordinate system and head position in the MEG scanner.

After the 20 practice trials, the main experiment was performed in one run, lasting about 17 minutes. Participants saw all stimuli twice, distributed over two lists. The lists differed with respect to face orientation: a specific face appeared upright in one list, and inverted in the other. Stimulus order was randomized separately within each list.

After presentation of a fixation cross for 500 ms, a face stimulus appeared for 1000 ms, with the nasion positioned at the centre of the screen. The face stimulus was again followed by the fixation cross, displayed after a jittered period between 1900ms and 2300ms (*M* = 2100ms). 500ms after the offset of the face stimulus, a tone was presented that served as “go” signal for a forced-choice button press, with which participants signalled whether the presented face was unknown or familiar. The response delay served to minimize overlap of visual processing with motor response preparation and execution. Allocation of the known/unknown responses to the left/right key of the response box was counterbalanced across participants. To minimise body movements, subjects responded with their left and right thumbs and the response box was positioned on their lap. The manual responses were taken to compute accuracy measures for each subject and condition. These data were analysed with a repeated measurement ANOVA with the factors Group (congenital prosopagnosics vs. controls), Familiarity (famous vs. unknown) and Orientation (upright vs. inverted). Note, that due to the delayed response the establishment of latencies was not a meaningful procedure.

After the MEG recording, all participants completed a test on upright versions of all pictures of the famous faces. The test procedure was similar to the BFFT [53]. While a picture was presented, participants were encouraged to provide information about the name of the person and/or report anything that they know about him/her. If they did not succeed with this, the name of the person was shown together with three unknown distractor names, and participants had to say which name was known to them. Upon identification of the famous person's name, they were again encouraged to retrieve any information about the person. If they did not know a face/name or could not provide any semantic information, the particular person was classified as unknown to them and excluded from further analysis. With this procedure, we were able to quantify which famous people were effectively known to the participants (especially for the prosopagnosic group), by visual cues, by verbal cues only or not at all.

### MEG recording and data processing

MEG signals were recorded using a 275-sensor whole-head MEG-system (Omega 275, CTF, VSM MedTech Ltd.) with first-order axial SQUID gradiometers (2 cm diameter, 5 cm baseline, 2.2 cm average inter-sensor spacing). Data were recorded continuously, with first-order gradient filtering at 274 sensors (one sensor turned off due to technical problems). Brain responses were sampled at 600 Hz and filtered online, with a frequency band-pass of 0–150 Hz. Recordings were further processed off-line using Brain Electrical Source Analysis (BESA®). Data were filtered using a 45 Hz low-pass and a 0.01 Hz high-pass filter. The averaging epoch was defined from 500 ms before stimulus onset to stimulus offset (−500 to 1000 ms), and data were baseline-corrected based on a 300 ms pre-stimulus interval.

During the averaging procedure, only those famous faces were included which according to the post-test were known by the participants, ensuring that the analyzed famous faces were actually known to our subjects. For the congenital prosopagnosics, mostly pictures of people were included whom they knew by name. On average, 50 trials (76%) remained in the condition of famous faces.

To evaluate the underlying neural activity, source-space activity was estimated for each time point in each condition and subject, using the least square minimum-norm-estimation (L2-MNE) method [54]. This inverse source modeling and the consecutive statistical analysis thereof was conducted with the Matlab-based EMEGS software (www.emegs.org). The L2-MNE is an inverse method allowing the reconstruction of distributed neural sources underlying the extracranially recorded event-related magnetic fields, without the necessity of a-priori assumptions regarding the number and possible locations of underlying neural generators. The L2-MNE is calculated by multiplying the pseudo-inverse of the so-called lead-field matrix (which describes the sensitivity of each sensor to the sources) with the averaged recorded data. Individual lead-field matrices were computed for each participant, based on information about the center and radius of a sphere fitting best to the digitized head shape, and the positions of the MEG sensors relative to the head. A spherical shell with 8 cm radius and with 350 evenly-distributed dipole locations served as distributed source model. At each dipole location, two perpendicular dipoles were positioned which were tangentially oriented to the spherical model.

**Figure 1:**
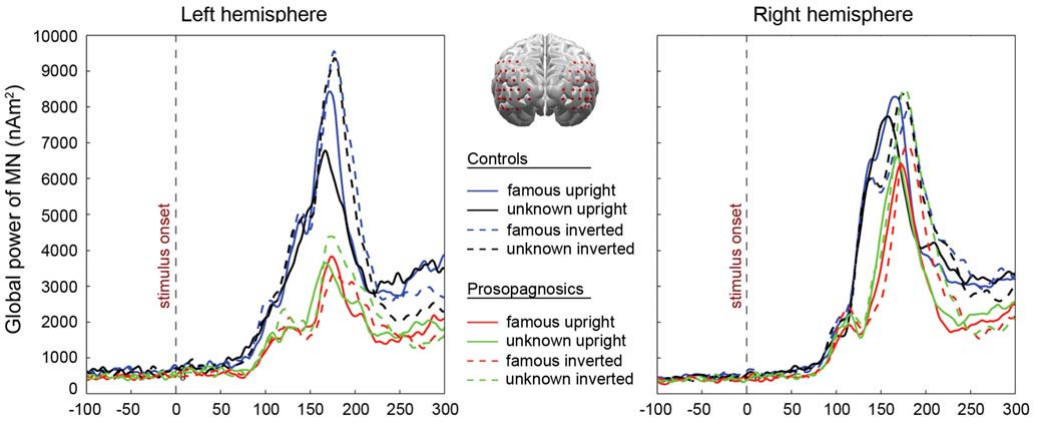
Left and right: Global Power of Minimum Norm Estimates separated by hemisphere, groups and conditions. Top middle: Sensors groups (red circles) selected for the analysis of brain responses regarding the M170 projected onto a standard brain model.

The results of the L2–MNE solution are source wave forms over time for each dipole location (vector length of the corresponding tangential dipoles). Visual inspection of the Global Power (mean squared activity across all sources and time points) of the Grand Mean (average across all subjects and conditions) of the L2–MNE solutions was used to establish the time intervals for the M170, ranging between 120 and 200 ms and reaching its peak around 170 ms (divided in subintervals of 10 ms, in order to describe the time course of activity in more detail). Homologous sensor groups in both hemispheres were established within this time interval and used for further statistical analysis. In each hemisphere, 34 sources (corresponding dipoles) within the occipitotemporal area were grouped together, such that homologous regions were achieved. The selection of representative dipoles was based on existing literature about estimated M170 generator locations [55]–[57] and on the averaged activity in the time interval ranging from 120 to 200 ms (see Figure 1, top middle). To investigate effects and interactions of experimental manipulations and group differences, repeated-measurement ANOVAs with the factors Group (congenital prosopagnosics vs. controls), Familiarity (famous vs. unknown), Orientation (upright vs. inverted), and Hemisphere (left vs. right) were calculated.

Based on the interval which differentiated best between groups, we calculated a laterality index (difference of left and right hemispheric activity divided by their sum) for each subject across all conditions. This index was correlated with the behavioral measures to recognize famous faces as familiar.

## Results

### Questionnaire and behavioural measures

Controls recognized on average 51/66 (77%) of the famous persons based on visual cues only, and an additional 10/66 (15%) based on verbal cues (61/66 in total; 92%). Individuals with congenital prosopagnosia recognized on average only 15/66 (23%) of the famous faces on visual cues only, but were able to identify 39/66 (59%) additional famous people by name (54/66 in total; 82%). Thus, prosopagnosics knew almost as many of the tested famous people as the controls, but compared to controls clearly not based on visual information alone. Based on both visual or verbal cues, on average controls failed to identify 5 out of 66 famous persons, and prosopagnosics 11 out of 66. When asked how they accomplished to correctly identify faces visually, all prosopagnosics reported that they recognized one or more characteristic features belonging to a particular face. The rate of identification based on visual cues alone is different between both groups (t = −6.016, df = 6; p = .001). This (expected) result clearly illustrates the deficit of our group of prosopagnosic individuals in face recognition, and corroborates findings from our recent study [9] in which most of the prosopagnosics tested here were neuropsychologically investigated.

Similarly, we found in the behavioural measures (see Table 1) a main effect for group (F(1, 12) = 17,945; p<.001). Controls classified more famous faces as known and unknown faces as unknown. In general, famous faces were recognized more often as familiar than unknown faces (F(1, 12) = 75,182; p< .001) and more errors were made in response to inverted compared to upright faces (F(1, 12) = 45,094; p<.001). These main effects were modulated by several interactions. There was only a marginal difference to respond more accurately to upright than inverted unknown faces (t(13) = 2.007; p = .066), but famous faces were categorized more correctly if presented upright compared to inverted (t(13) = 4.380; p = .001). This resulted in the interaction of the factors Familiarity and Orientation (F(1, 12) = 8.621; p = .012). An interaction of Familiarity and Group (F(1, 12) = 9.664; p = .009) arose, because the two groups did not show a difference in classifying unknown faces as unknown (t(12) = .024; n.s.), but controls recognized more famous faces as known (t(12) = 3.895; p = .002). Controls made more correct classifications in response to upright compared to inverted faces (t(6) = 6.170; p = .001) as did persons with congenital prosopagnosia (t(6) = 2.808; p = .031). Because this difference was more strongly expressed in controls, this led to the significant interaction of Group and Orientation F(12) = 13.782; p = .003).

**Table 1:** Number of correct behavioural responses in all conditions and laterality index

Subject	Correct responses				Laterality Index
	famous faces		unknown faces		
	upright	inverted	upright	inverted	
**CPs**					
XG	14	1	65	64	.04
BT	7	0	65	61	−.23
GH	10	14	66	64	−.29
XS	18	5	64	61	−.23
MH	37	15	49	59	−.25
LO	4	4	48	50	−.23
KA	11	5	65	65	−.07
***Mean (SD)***	*14.4 (10.9)*	*6.3 (5.9)*	*60.3 (8)*	*60.6 (5.1)*	*−.18 (.12)*
**Controls**					
***Mean (SD)***	*46.9 (16.8)*	*26.6 (18.2)*	*63.9 (2.3)*	*56.9 (7.3)*	*−.01 (.10)*

### Magnetoencephalographic Data

The Global Power of the overall estimated neural activity (L2-MNE) across all conditions for both subject groups (see Figure 1, left and right) shows an early, small peak around 100 ms and a broader, much stronger component peaking around 170 ms. Since previous research suggests a major role of the N170/M170 in face processing, we focused on the processes underlying its formation. All results reported here will thus refer to this component. Given that there were no significant effects regarding the latency of the M170, only amplitude differences are reported.

**Figure 2:**
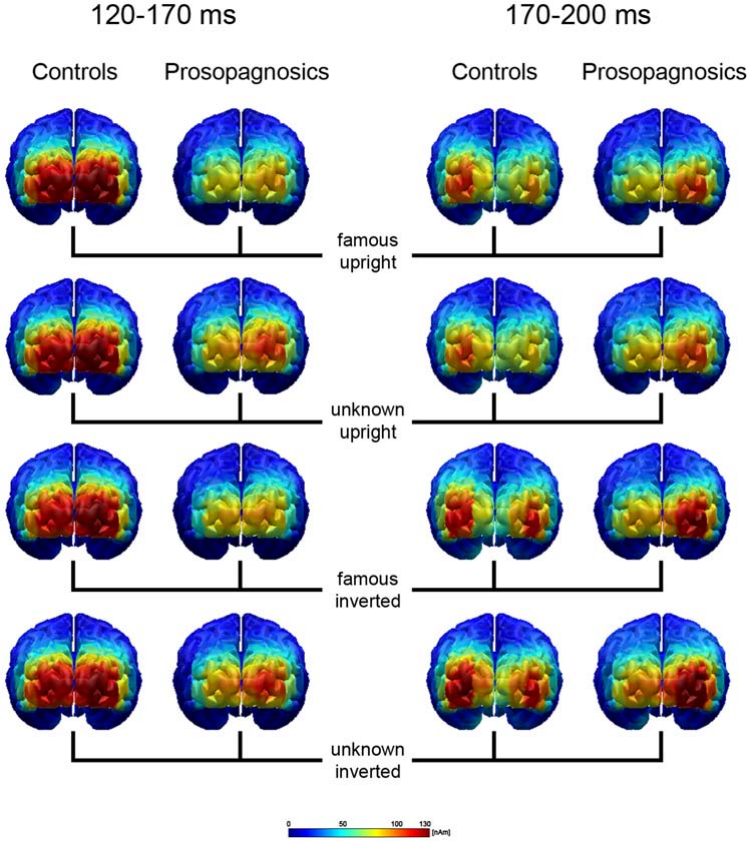
Mapping of Minimum Norm Estimates for the time interval of the M170 (displayed in an early and a late interval) separated by groups and conditions.

The estimated neural activity in the M170 time interval is much less pronounced in the group of individuals with congenital prosopagnosia, and seems particularly reduced in the left hemisphere. Figure 2 displays the L2-MNE topography of activity from an occipital viewpoint, for all conditions and time intervals. Two intervals (120–170 ms; 170–200 ms) were chosen to display the time course of activity regarding the rising and falling slope of the M170 in somewhat more detail.

**Figure 3:**
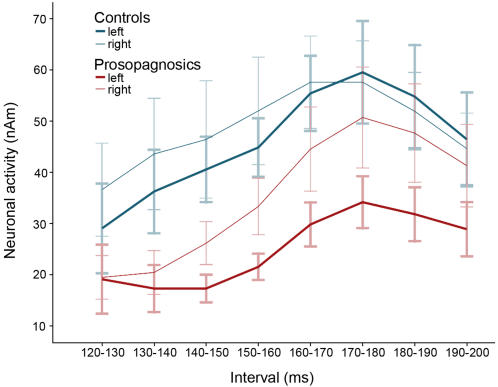
Time course of activity for the M170 averaged across conditions and displayed separately for groups and hemispheres (error bars represent one standard error).

The descriptive impressions were confirmed by statistical analyses. For the selected interval of 120–200 ms, we found a main effect for Group (F(1, 12) = 4.88; p< .05), with overall stronger activity in controls than in congenital prosopagnosics. There was also a main effect of Orientation (F (1,12) = 5.056; p<.05), with higher activity upon inverted than upright faces. Activity was generally more expressed in the right hemisphere (main effect Hemisphere: F(1,12) = 6.241; p = .05). Given that we were interested in the time course of activity, we divided the interval from 120 to 200 ms in 10 ms steps (figure 3). The overall amount of activity differed between these intervals (main effect Time: F(7, 84) = 6.804; p<.001; Greenhouse-Geisser corrected p<.01), with activity increasing towards the M170 interval, and declining afterwards. The increase in activity from early intervals (120–130) to the intervals between 160 and 180 ms, was marginally significant (both p <.10; Bonferroni corrected). From the peak around 170 ms (time interval between 170–190 ms), activity significantly decreased in the 190–200 ms interval (both p<.05; Bonferroni corrected). There was no main effect of Familiarity (F(1, 12) = .513; n.s.).

These main effects were modified by several interactions. The main effect of Orientation was modified by Time (F(7, 84) = 12.741; p<.001; Greenhouse-Geisser corrected p<.001) and, as expected, by Familiarity (F(1, 12) = 12.719; p = .004). In addition, there was a three-way-interaction of Orientation×Familiarity×Time (F(7, 84) = 4.582; p<.001; Greenhouse-Geisser corrected p<.01). Face inversion had an early and long lasting impact on brain response, which reversed over time. While the effect was only marginal between 140 and 150 ms (F(1,12) = 3.68; p = .079), it was highly significant between 150 and 160 ms (F(1,12) = 10.219; p = .008), with higher activity upon upright faces. This pattern of activity reversed between 170 and 180 ms (F(1,12) = 5.663; p = .035), and later (180–190: F(1,12) = 31.647; p<.001; 190–200: F(1,12) = 25.567; p<.001). Thus, in the typical time range of the M170 and well after, as expected, we found stronger brain activity evoked by inverted than by upright faces. The interaction of Orientation and Familiarity was reliable between 160 and 190 ms (for 160–170: F(1, 12) = 15.08; p = .002; for 170–180: F(1, 12) = 19.162; p = .001; for 180–190: F(1, 12) = 6.001; p = .031). Again, this is the typical time interval of the M170. Averaged across these intervals, the enhanced activity for inverted compared to upright faces was significant only for unknown faces (t(13) = 4.011; p = .001). The numerical difference in the same direction, for known faces, was not reliable (t(13) = .417; n.s.).

Besides the reduced overall activity for prosopagnosics, we found no interactions of Group with Familiarity and Orientation. There was, however, a significant three-way-interaction of Group×Hemisphere×Time (F(7, 84) = 3.1; p = .006; Greenhouse-Geisser corrected: p = .054), which showed that the interaction of Group×Hemisphere was restricted to the intervals between 170 and 200 ms (170–180: F(1, 12) = 6.241; p = .028; 180–190: F(1, 12) = 6.117, p = .015; 190–200: F(1, 12) = 7.758; p = .016). Averaged across these intervals, the controls showed no difference between hemispheres (t(6) = .660; n.s.). However, participants with congenital prosopagnosia displayed significantly less activity in the left compared to the right hemisphere (t(6) = −2.910; p = .027). Thus, a lateralisation effect was only seen in congenital prosopagnosics, with reduced left-hemispheric activity. The three-way interaction of Group×Hemisphere×Time is illustrated in Figure 3.

The time interval between 170 and 200 ms served to calculate the laterality index of the estimated neural activity for each subject across conditions. As expected from the results above and shown in Figure 3, controls showed no hemispheric difference (i.e. the index did not differ from zero (t(6) = −.248; n.s.) while congenital prosopagnosics displayed less left than right hemispheric activity (i.e. the index is smaller than zero t(6) = −4.027; p = .007). There was no significant correlation of the laterality index and the behavioural measures.

## Discussion

We set out to investigate the neural correlates of congenital prosopagnosia by tracking cortical activity using magnetoencephalography with high temporal accuracy. We manipulated two factors that are sensitive to face processing mechanisms and to the face processing impairment itself. By varying face familiarity, we investigated the key diagnostic criterion for prosopagnosia, that is, not being able to recognize a familiar person from face. We also manipulated the orientation of the stimuli. Previous studies suggested that congenital prosopagnosia results from the impaired configural processing of faces, which is usually investigated by contrasting the processing of upright and inverted pictures.

The behavioural measures confirmed our expectations and corroborated earlier research. Controls recognized more famous faces than prosopagnosics and recognition performance in both groups was hampered by face inversion. More importantly, controls had a greater advantage than prosopagnosics if faces were presented in upright compared to inverted orientation. This corresponds to a reduced or missing face inversion effect already reported by other authors [13], [26]–[27]. Thus, the behavioural data support the assumption of impaired configural/holistic/global processing in congenital prosopagnosia.

A brief summary of the magnetoencephalographic data goes as follows. First, over occipitotemporal areas we observed an overall increase of activity from early time windows to time windows that are typical for the M170, followed by a decrease. Thus, peak activity was within the expected time frame for the M170. Second, inverted faces were associated with higher neural activity, in particular during the M170 time windows and beyond. Between 160 an 190 ms, this inversion effect was larger for unknown than for known faces. Third, there was more right-hemispheric involvement overall. For participants with congenital prosopagnosia, this held for all time windows, for control subjects, there was equal activation in the right and left hemisphere in the 170–200 ms interval. Finally, face stimuli clearly induced less overall neural activity in prosopagnosics compared to control subjects. We will discuss these findings and their implications in turn.

For the M170, our main component of interest, there was more activity in response to inverted than to upright faces, and this was more strongly expressed when faces were unfamiliar. This is in line with our predictions, given that we expected no main effect of familiarity but an interaction with orientation. These results corroborate findings for unimpaired face perception. As argued earlier, effects of orientation bear on the issue of configural vs. featural processing. Face inversion is thought to be particularly harmful for configural processing [58]–[59]. It is also assumed that configural processing is specifically relevant to the recognition of familiar faces [60]–[61]. In support of these arguments, we found more strongly expressed neural activity in the M170 interval in response to inverted unfamiliar than to inverted familiar faces. Note, that we had expected different inversion effects for prosopagnosics and control subjects, but the relevant interaction was not present in the data.

Second, we replicated the classical finding of stronger right than left hemispheric activity in face processing, as shown in lesion studies (for an overview see ref. [3]), and in imaging studies [42]–[44], [62]. The right hemisphere is supposed to process facial information on a more holistic basis, whereas the left hemisphere seems to be more dedicated to the analysis of facial features [63]. More specifically, Rossion and colleagues [64] found that the right fusiform gyrus is involved in holistic processing whereas the left fusiform gyrus subserves feature-based processing.

Third, we observed the expected asymmetry between prosopagnosic and unimpaired subjects with respect to overall activity. In our effort to track the neuronal activity with high accuracy in time, we found bilaterally less activity for the congenital prosopagnosics in the initial phase of the M170, followed by an even larger reduction for the left hemisphere. We had expected a larger right-hemispheric reduction, based on recent neuroanatomical findings by Behrmann and colleagues [49], as well as on assumptions about impaired configural processing in congenital prosopagnosia, which is taken to be subserved by the right hemisphere [64]–[65], [for an overview of other hypothesis see ref. 66]. This assumption was also supported by the behavioural data reported above.

However, even though the reduction of left hemispheric activity in congenital prosopagnosia seems puzzling, there is already indirect evidence for a reduced involvement of the left hemisphere in the literature. First, and although Avidan *et al*. [45] themselves reported no reduction of FFA activity measured with fMRI for their four congenital prosopagnosics, Bentin and colleagues [18], pointed out that closer inspection of the data revealed that “… left FFA selectivity was not present in the CP group, whereas both right and left FFA activity was robust in the control group.” (p. 144). This can be seen clearly in Figure 1 of their article [45, p. 1152] on both, the averaged activation maps as well as on a single case basis. Note also, that in a study of Bentin and coauthors [36], the N170 showed the largest difference between controls and their prosopagnosic subject Y.T. over the left hemisphere (see Figure 1, p. 826) even though this effect was also not discussed. Our data substantiate these observations. In addition, we were able to show that the reduced left-hemispheric activity appears early after stimulus onset.

How can this result be reconciled with the current literature? A possible argument is based on brain-asymmetrical processing of configural and featural information. It is assumed that congenital prosopagnosics excessively use a feature-based approach in order to compensate for their impairment in configural processing [26]. As we know from other domains of mental processing, increasing expertise or use may lead to decreased activity in some brain regions [67] signalling the establishment of more lean and efficient processing. Thus, reduced left hemispheric activity might be the result of expertise and overuse of featural information in face processing in prosopagnosics.

In conclusion, we feel safe to say that congenital prosopagnosia is related to an overall reduced activity in occipitotemporal areas which is especially prominent in the left hemisphere, most likely caused by the generators of the M170 (N170). This was evidenced by three different research groups with different subjects and methods, even though it was not expected and at times not even mentioned. Sometimes it seems necessary to search for a lost key even in regions with little light.
